# Metabolic Profiling of Brain Tissue and Brain‐Derived Extracellular Vesicles in Alzheimer's Disease

**DOI:** 10.1002/jev2.70043

**Published:** 2025-02-03

**Authors:** Patricia Hernandez, Elisabeth Rackles, Oihane E. Alboniga, Pablo Martínez‐Lage, Emma N. Camacho, Arantza Onaindia, Manuel Fernandez, Ana Talamillo, Juan M. Falcon‐Perez

**Affiliations:** ^1^ Exosomes Laboratory, Center for Cooperative Research in Biosciences (CIC bioGUNE) Basque Research and Technology Alliance (BRTA) Derio, Bizkaia Spain; ^2^ Metabolomics Platform, Center for Cooperative Research in Biosciences (CIC bioGUNE) Basque Research and Technology Alliance (BRTA) Derio, Bizkaia Spain; ^3^ Center for Research and Advanced Therapies CITA‐Alzheimer Foundation Gipuzkoa Spain; ^4^ Anatomic Pathology Araba University Hospital Vitoria‐Gazteiz Alava Spain; ^5^ Bioaraba Health Research Institute Oncohaematology Research Group Vitoria‐Gasteiz Spain; ^6^ Pathology Department Osakidetza Basque Health Service Araba University Hospital Vitoria‐Gasteiz Spain; ^7^ Neurological Department Hospital Universitario Cruces (HUC) Barakaldo Spain; ^8^ Neuroscience Department Universidad del País Vasco (UPV‐EHU) Leioa Spain; ^9^ Biomedical Research Centre of Hepatic and Digestive Diseases (CIBERehd) Carlos III Health Institute (ISCIII) Madrid Spain; ^10^ IKERBASQUE Basque Foundation for Science Bilbao, Bizkaia Spain

**Keywords:** Alzheimer's disease, brain, extracellular vesicles, mass spectrometry, metabolomics

## Abstract

Alzheimer´s disease (AD) is the most frequent neurodegenerative disorder in the world and is characterised by the loss of memory and other cognitive functions. Metabolic changes associated with AD are important players in the development of the disease. However, the mechanism underlying these changes is still unknown. Extracellular vesicles (EVs) are nano‐sized particles that play an important role in regulating pathophysiological processes and are a non‐invasive manner to obtain information of the cell that is secreting them. The analysis of brain‐derived EVs (bdEVs) will provide new insights in the metabolic processes associated with AD. To characterize bdEVs in AD, we optimised a method to isolate them from tissue of different brain regions, obtaining the highest enrichment in isolations from the temporal cortex. We performed unbiased untargeted metabolomics analysis on post‐mortem human temporal cortex tissue and bdEVs from the same region of AD patients and healthy controls. Both, univariate and multivariate statistical analysis were used to determine the metabolites that influence the separation between AD patients and controls. Interestingly, a clear separation between control and AD groups was obtained with bdEVs, which allowed to select 12 relevant features by a validated PLS‐DA model. Furthermore, comparison of tissue and bdEVs identified 68 common features. The pathway enrichment analysis of the common metabolites showed that the alanine, aspartate and glutamate pathway and the arginine, phenylalanine, tyrosine pathway were the most significant ones in the separation between the AD patients and controls. The phenylalanine, tyrosine and tryptophan pathway, still had a very high influence in the separation between groups, albeit not significant. Notably, some metabolites were identified for the first time in bdEVs. For example, the N‐acetyl aspartic acid (NAA) metabolite present in bdEVs was suitable to differentiate AD patients from healthy controls. Furthermore, the analysis of the hippocampus, midbrain, temporal and entorhinal cortex and their respective bdEVs indicated that the metabolic profiles of different brain areas were distinct and showed some correlation between the metabolome of the tissue and its respective bdEVs. Thus, our study highlights the potential of bdEVs to understand the metabolic fingerprint associated with AD and their potential use as diagnostic and therapeutic targets.

## 1 Introduction

Alzheimer´s disease (AD) is the most common form of dementia worldwide, representing up to 70% of all cases. It is manifested by the progressive loss of memory, cognitive deterioration, impairment of daily activities and behavioural disturbances. The prevalence rates for this disease are expected to increase over the next decades, with an estimation of 153 million individuals affected worldwide by 2050 (Nichols et al. 2022). This complex and multifactorial neurodegenerative disorder is pathophysiologically characterised by the deposition of extracellular amyloid‐β plaques, intracellular neurofibrillary tangles (NFTs) containing aggregated tau protein and loss of synaptic connections within selective brain regions, mainly in the frontal and temporal lobes (Glenner and Wong 1984; Masters et al. 1985; Goedert, Spillantini, and Crowther 1991; Murphy and Levine 2010). Currently, there is no cure for AD and, despite promising drugs recently approved, the treatments are restricted to the management of symptoms. The diagnosis of AD is usually established based on the clinical symptoms. However, the onset of the disease begins many years before. Therefore, it is important to identify precise biomarkers involved in the occurrence and progression of the disease before the manifestation of the clinical symptoms. Over the years, a great effort has been undertaken in this sense. Potential methods for early diagnosis of AD include identifying amyloid by PET scanning, and measuring the Aβ_42_ peptide isoform, the Aβ_42/40_ ratio, total tau (t‐tau) and phosphorylated tau (p‐tau181, p‐tau217, p‐tau231) peptides in cerebrospinal fluid (CSF) and plasma samples of AD patients (Chapleau et al. 2022; Janelidze et al. 2016; Wattmo, Blennow, and Hansson 2020; Ashton et al. 2022).

Extracellular vesicles (EVs) are nano‐sized particles enclosed by a phospholipid bilayer membrane that are secreted by all cell types and are present in biological fluids including serum, plasma, urine, saliva and CSF (Liu and Wang 2023). Research during the last years suggests that EVs have pleiotropic roles in the development of AD Thus, several studies in human and mouse indicate that EVs can stimulate the aggregation of Aβ_1‐42_, mediate the secretion and propagation of Tau pathology and contribute to neuroinflammation (Saman et al. 2012; Song et al. 2020; Wang et al. 2017; Ruan et al. 2021). On the other hand, EVs from different origins can mitigate the disease as shown by their ability to reduce amyloid‐β deposition, oxidative stress, synaptic damage and to transfer neuroprotection between cells (Katsuda et al. 2013; De Godoy et al. 2018; Apodaca et al. 2021; Chen et al. 2021).

Albeit the advances in AD knowledge in the last decades, the molecular mechanisms behind the onset and progression are still unknown. Metabolomics, which is a powerful approach to analyse complex dynamic metabolic changes, can provide new insights into the biological pathways associated with AD. Interestingly, one of the first changes described in AD before the manifestation of the clinical symptoms is perturbations in metabolic pathways (Meles et al. 2017; Pagani et al. 2017). Several metabolomic studies, using serum, plasma, CSF, urine or saliva samples have demonstrated the impact of AD pathogenesis on metabolism (Yin et al. 2023). Some of the main metabolic changes are reduced glucose metabolism, altered glutamate and glutamine levels, impaired lipid metabolism, mitochondrial dysfunction and impaired neurotransmitter and amino acid metabolism (Ogawa et al. 1996; Mullins, Reiter, and Kapogiannis 2018; Shao et al. 2020; Cheng and Bai 2018; Wang et al. 2014; Peña‐Bautista et al. 2022; Pomara et al. 1992; Jiménez‐Jiménez et al. 1998; Kaiser et al. 2010; Zhang et al. 2020; Liu et al. 2021; Akyol et al. 2021; Griffin and Bradshaw 2017). Similarly, studies on brain tissue from different regions also indicated an association of AD with altered metabolomic regulation (Liu et al. 2021; Paglia et al. 2016; Ambeskovic et al. 2023; Kim et al. 2019; Snowden et al. 2019). Recent untargeted metabolomics on hippocampus samples showed that the most significant upregulated pathways in AD were arginine and proline metabolism and pentose phosphate pathways, whereas alanine, aspartate and glutamate metabolism, pyruvate metabolism, glycolysis/gluconeogenesis, pyrimidine metabolism and aminoacyl‐tRNA biosynthesis were downregulated (Maffioli et al. 2022). Recently, an increasing number of studies have focused on the participation of EVs in metabolism, mostly focusing on cancer. The characterization of metabolites that are present in brain‐derived EVs (bdEVs) in AD and the comparison with brain tissue and biofluid samples would help to interpret the pathophysiology and heterogeneity of this disease. However, few studies have successfully explored the implication of the bdEVs’ metabolism in AD pathogenesis. A lipidomic analysis of EVs isolated from frontal cortex tissue identified altered glycerophospholipids and sphingolipids levels in AD patients (Su et al. 2021). In contrast, in a recent analysis of serum‐ and EV‐derived metabolites from patients with AD, mild cognitive impairment, and healthy controls showed the challenge to detect small metabolites in EVs samples. The study did not identify significant EV‐derived metabolites that could differentiate patients from healthy individuals (Nielsen et al. 2021).

In this study, we optimised a method to isolate bdEVs from different post‐mortem brain regions that allowed us to apply successful metabolomic approaches. We performed untargeted metabolomics of the polar metabolome by ultra‐high performance liquid chromatography coupled to a hybrid quadruple‐time of flight mass spectrometer (UHPLC‐Q‐ToF‐MS) to analyse and compare tissue from frozen temporal cortex and bdEVs from the same region of clinically characterised AD patients and age‐matched controls to identify differences. Furthermore, different brain regions and their corresponding bdEVs were also analysed by untargeted metabolomics to study metabolic brain heterogeneity. Our findings highlight the importance of bdEVs to better understand the altered metabolism in AD pathology as well as their metabolic correlation with their parental brain tissue.

## 2 Materials and methods

### 2.1 Reagent and Solutions

All reagents and solutions used in this work are included in the Supporting Information with detailed information.

### 2.2 Study Cohort

Post‐mortem human brain tissues, stored at –80°C, were obtained from the Basque Biobank (Neurological Tissue Bank, BIOEF). Temporal cortex tissues of 14 AD subjects (*n* = 7 male and *n* = 7 female; mean age 74.6 ± SD 10.8 years) displaying Braak stages III‐V and 10 age‐matched CTRL subjects (*n* = 7 male and *n* = 3 female; mean age 73.8 ± SD 15.5 years) with no evidence of dementia were included in this study (Table 1). The relevant clinical pathological information is provided in Table S1. Ethical approval number of the study is PI+CES‐BIEF 2021‐14, and all procedures were approved by the Research Ethics Committee with Medicines in the Basque Country (CEIm‐E) and conducted in accordance with **L**aw 14/2007, on Biomedical Research; Ethical Principles of the Declaration of Helsinki (2008) and Royal Decree 1716/2011 of 18 November.

**TABLE 1:** Sample information of the cohort analysed in the study.

Case	Age	Sex	ABC score	Braak stage	PMI (h)
AD2	86	F	ND	ND	8
AD4	52	F	ND	IV	14
AD7	59	F	ND	VI	36
AD24	89	M	A1B3C1	V or VI	37
AD34	77	M	A1B3C2/3	V or VI	14
AD35	76	M	A1B2C1/2	III or IV	17
AD36	69	M	A3B3C2/3	V or VI	22
AD37	75	F	A1B3C2	V or VI	21
AD38	79	M	A1B3C2	V or VI	23
AD40	69	M	A1B2C1	III or IV	16
AD41	84	F	A1B3C2	V or VI	10
AD42	77	F	A3B2C2/3	III or IV	23
AD43	65	M	A3B3C1	V or VI	18
AD44	86	F	A1B3C2	V or VI	24
AD45	87	F	A1/2B2C2	III or IV	3
CTRL1	68	M	—	—	23
CTRL2	100	M	—	—	1
CTRL3	49	F	—	—	24
CTRL4	52	F	—	—	19
CTRL5	76	M	—	—	7
CTRL6	80	M	—	—	18
CTRL7	67	M	—	—	36
CTRL8	78	F	—	—	24
CTRL9	89	M	—	—	3
CTRL10	79	M	—	—	21

*Note*: Biobank ABC score: A, ThaI Phase for β‐amyloid plaques; B, Braak stage of neurofibrillary tangles; C, CERAD neuritic plaque score.

Abbreviations: AD, Alzheimer's disease; CTRL, healthy control; M, male; F, female; PMI, post‐mortem interval in hours; ND, no data available.

### 2.3 Sample Preparation

#### 2.3.1 EV Isolation From Human Brain Tissue

For EV isolation from frozen human brain tissue, a protocol was adapted from the protocol established previously by Huang et al. (2020). Briefly, approximately 1 g of the hippocampus, temporal cortex, midbrain and entorhinal cortex tissues were manually dissected on ice into smaller sections (1, 2 cm long and 2–3 mm wide). Brain sections were incubated in a water bath at 37°C for 20 min in the digestion buffer Hibernate‐E medium containing 50 U/mg collagenase type 3 and mixed after 10 and 15 min of incubation. For stopping the tissue digestion, 7× inhibition buffer, which contained PhosSTOP phosphatase inhibitor (PS) and complete protease inhibitor (PI) in Dulbecco's phosphate buffered saline previously filtered with a 0.10 µm sterile vacuum filtration system (Millipore), was added to the cold brain sections to a final concentration of 1×. The dissociated tissue was centrifuged first at 300 × *g*, 10 min at 4°C, and then at 2000 × *g* for 15 min at 4°C to obtain the cell‐free supernatant. Then the supernatant was centrifuged at 10,000 × *g* for 30 min at 4°C using the TLA‐110 rotor (Beckman Coulter) to obtain the small EV (sEV)‐containing supernatant. The sEV‐containing and cell‐free supernatants was concentrated with a 100 kDa MWCO Pierce Protein Concentrator PES (Thermo Scientific) from 6 mL to 300 µL by centrifugation at 3900 × *g* at 4°C. From the concentrated sample, 200 µL were fractionated using an in‐house size exclusion chromatography (SEC) for which Sepharose CL‐2B SEC columns had been pre‐rinsed twice with 2 mL of 0.10 µm filtered DPBS. From the SEC, 10 fractions containing 200 µL and two final fractions of 1 mL volume were collected by elution with 0.10 µm filtered DPBS. The first two fractions eluted was considered the void volume; fractions 3–5 (F3‐5) and 8–10 (F8‐10) were EV‐ and protein‐enriched, respectively. A total of 150 µL of each F3‐5 and F8‐10 were pooled and 550 µL of 0.10 µm filtered DPBS were added to each sample pool. To further purify and concentrate EVs and proteins, the pooled fractions were spun for 70 min at 110,000 × *g* at 4°C using the TLA‐120.2 rotor (Beckman Coulter). The supernatant was removed, and the pellets were resuspended in 100 µL of 0.10 µm filtered DPBS (Figure S1). EV and protein‐enriched samples were stored until analysis at –80°C and repeated freeze‐thaw cycles were avoided.

#### 2.3.2 Human Brain Tissue Preparation and Protein Quantification

For the homogenization and protein quantification of human brain tissue samples (including the hippocampus, temporal cortex, midbrain and entorhinal cortex regions used for cortex selection; and the temporal cortex used for unbiased untargeted metabolomics analysis), approximately 50 mg pieces of each tissue were collected into 2 mL Precellys Tissue homogenization tubes with 500 mg of 1.4 mm zirconium oxide beads and 0.10 µm filtered DPBS solution containing PS and PI. The tissue was homogenised following one cycle of homogenization at 6 m/s for 40 s using a FastPrep‐24 homogenizer (MP Biomedicals). The homogenate was spun at 15,000 × *g* for 1 min at 4°C. From the tissue pieces used for EV isolation, the pellet collected after centrifugation at 300 × *g* was also homogenised with 500 mg of beads and DPBS with PS and PI. The supernatant was spun at 15,000 × *g* for 15 min at 4°C. The supernatant of both cases was collected and nominated as ‘Brain homogenate’ (BH) and ‘Brain homogenate after treatment with collagenase’ (BHC), respectively (Figure S1).

After centrifugation at 10,000 × *g* for 30 min at 4°C with the TLA‐110 rotor (Beckman Coulter), the pellet was resuspended in 100 µL DPBS with PI and PS and was nominated as ‘P10k’. After sample concentration with a 100 kDa MWCO protein concentrator (Thermo Scientific), supernatants were also collected and nominated as ‘S10k’ (Figure S1). All samples were stored at –80°C and then used for Western blotting analysis.

Protein content in tissue homogenates and EV suspensions was determined by Bradford protein assay according to the manufacturer's instructions.

### 2.4 Metabolite Extraction Procedure—Biphasic Extraction

Tissue and EVs isolated from tissue were treated to extract as many metabolites as possible. Due to the scarce amount of sample and the purpose of maximising the extraction of metabolites from these samples, a biphasic extraction was performed. Briefly, 50 mg of frozen tissue was directly weighted in 2 mL Precellys Tissue homogenization tubes and 500 mg of 1.4 mm zirconium oxide beads were added. Then, 500 µL of a mixture of cold MeOH: H2O (50:50) was added and homogenization was done in a FastPrep‐24 (MP Biomedicals). Afterwards, 400 µL of homogenised sample was transferred to an Eppendorf tube and 400 µL of cold chloroform was added. In the case of EVs the extraction protocol is comparable with tissue where 100 µL of cold MeOH were added to 100 µL of isolated‐EVs. After vortex mixing, 200 µL of cold chloroform were added maintaining the same extraction proportions as in tissue of 1:1:2 (MeOH: H2O: chloroform).

In both cases, samples were incubated in a Thermomixer compact (Eppendorf Iberica S.L.U., Madrid, Spain) for 1 h, at 4°C and 1400 rpm, and then centrifuged for 30 min at 4°C and 14,000 rpm in a Mikro 220R centrifuge (HettichLab, Tuttlingen, Germany). 300 and 160 µL of the non‐polar phase in tissue and EVs, respectively were collected, evaporated to dryness and stored at –80°C until further analysis. For the polar phase included in this study, 300 and 170 µL were collected, evaporated to dryness and resuspended in 300 and 130 µL of acetonitrile/water (ACN/H_2_O; 60/40; v/v) to analyse the polar fraction of the metabolome in tissue and EVs, respectively. All samples were centrifuged for 15 min at 4°C and 14,000 rpm to remove any suspended particle. Finally, 100 µL were transferred to a chromatographic vial to be injected into the UHPLC‐ToF‐MS system and 40 µL of each resuspended sample were mixed to prepare the quality control (QC) sample for tissue (QC_T_) or EVs (QC_EV_). Then, 350 µL of each QC were mixed to prepare the study QC that contains same amounts of all samples. To remove any compound or metabolite coming from solvents blanks samples were prepared. Solvent blank (ACN/H_2_O; 60/40; v/v), and experimental blank, which was prepared as a sample but without any biological material.

### 2.5 Characterization of the Extracellular Vesicles Preparations

bdEV characterization was performed using three different techniques: Western blotting, cryo‐electron microscopy (cryo‐EM), and nanoparticle‐tracking analysis (NTA). Western blotting analysis was performed under non‐reducing conditions. Samples, in LDS Sample Buffer, 1X, were denatured by incubation for 5 min at 37°C, 10 min at 65°C, 15 min at 95°C, and centrifuged at 20,000 × *g* (13,000 rpm) for 1 min. The supernatant was electrophoresed on NuPAGE 4%–12% Bis‐Tris pre‐casted gels. Proteins from the electrophoresis were transferred to a PVDF membrane by the Invitrogen XCell Surelock Mini Cell protein gel electrophoresis system (Thermo Fisher Scientific). The membrane was blocked in TPBS with 5% milk powder and incubated overnight at 4°C with the primary antibody (1:1000 in TPBS with 5% BSA) (see Supporting Information). Then, it was incubated with a secondary HRP‐conjugated antibody (1:6000 in blocking buffer) for 45 min at room temperature (see Supporting Information). Chemiluminescent bands were detected with Clarity Western ECL Substrate (Bio‐Rad) on an ImageQuant LAS 4000 imager (GE Healthcare). For cryo‐EM analysis, EV preparations derived from temporal cortex samples were vitrified using glow‐discharged holey carbon grids (blotted at 95% humidity) from Quantifoil, and liquid ethane (Vitrobot system; Maastricht Instruments). After vitrification, samples were imaged using a JEM‐2200FS/CR transmission cryo‐EM (JEOL) at liquid nitrogen temperature and at an acceleration voltage of 200 kV. To study size distribution, SEC‐fractions obtained from temporal cortex samples were analysed by NTA. A NanoSight NanoSight LM10 system (Malvern, UK) and particle‐tracking software (version 3.4) was used. For each fraction, three videos of 60 s each were taken at camera level 9 with a constant detection threshold of 5. All data obtained from these experiments are available in the EV‐TRACK knowledgebase (https://www.evtrack.org/index.php) with the EV‐TRACK ID: EV240046 (Van Deun et al. 2017).

### 2.6 Untargeted Metabolomics UHPLC‐ToF‐MS Analysis

Samples were analysed in an ultrahigh performance liquid chromatography system (UHPLC, Acquity, Waters Inc., Manchester, UK) coupled to a hybrid quadrupole‐time of flight mass spectrometer (Q‐ToF MS, SYNAPT G2, Waters Inc.). Chromatographic separation was performed by injecting 2 µL in a 2.1 × 100 mm, 1.7 µm BEH Amide column (Waters Inc.), thermostated at 40°C and 0.250 mL/min flow rate. A binary solvent system consisting of 1 mM of ammonium formate, and 0.5% formic acid in water (phase A—aqueous phase), and 1 mM of ammonium formate, 0.5% formic acid, and 5% water in acetonitrile (phase B—organic phase) was used for the elution at positive and negative ionization modes. The gradient started at 20% of A and reached 99.9% A at minute 9. It was maintained for 1 min and then starting conditions were recovered by minute 10.1. Initial conditions were kept for re‐equilibration until a total run time of 13 min.

The mass spectra data were acquired in positive and negative electrospray ionization modes in full scan (50–1200 Da). The cone voltage was 25 V, and the capillary voltages were 0.25 and 0.50 kV for positive and negative, respectively. The other source parameters were kept constant in all the experiments: source temperature 120°C, desolvation gas (nitrogen) temperature 450°C at a flow of 600 L/h, and cone gas (nitrogen) flow 5 L/h. A 2 ng/mL leucine‐enkephalin solution in H_2_O/ACN/formic acid (49.9/50/0.1) was infused at 10 µL/min and used for a lock mass which was measured each 36 s for 0.5 s. Spectral peaks were automatically corrected for deviations in the lock mass. The data were acquired using the MassLynx V4.1 from Waters. Blank samples were injected at the beginning and at the end of the sequence. A polar mixture which contains standards that covers the total chromatogram was injected to control mass accuracy and chromatographic performance. The QC sample was analysed at the beginning (after blanks) to condition the LC system (conditioning QCs) and then every six randomised sample to avoid any time related effects. These last QCs (analytical QCs) were used to assess reproducibility and stability of the system. QC_T_ and QC_EV_ were also injected after the QC every six randomised samples. The last QCs (QC, QC_EV_ and QC_T_) were also acquired in data independent acquisition (DIA) mode to get fragmentation information to further identify significant and relevant metabolites. The parameters setting of DIA method, which contained a function of MS and functions of MS/MS at different voltages, were the same for MS as the previous full scan and for MS/MS were mass range from 50 to 1200 Da, 0.2 s scan time and fixed collision energy of 10, 20, 30 and 40 V.

### 2.7 Data Deconvolution and Preprocessing

Untargeted metabolomics analysis was firstly checked using the MassLynx V4.1 to determine the chromatographic performance quality and the system mass accuracy as well as QC injection reproducibility and pressure stability. Then, raw data was processed with MS‐Dial version 4.9.221218 (https://systemsomicslab.github.io/compms/msdial/main.html) to perform the deconvolution process and alignment, and to obtain the matrix with the retention time (RT), mass‐to‐charge ratio (*m/z)* and areas of each feature (defined as *m/z*—RT). In short, MS‐Dial parameters were selected based on the LC‐MS system performance. Afterwards, features coming from blanks were removed. Then, R Statistical Software (v4.1.2; R Core Team (2021)) (R Core Team 2014) was used to perform the median fold change normalization to correct and normalise differences in tissue weights or sample management. Then, those features with percentage of coefficient of variation (% CV) greater than 30 in QC samples were removed and the final filtered matrix was processed with SIMCA‐P version 13.0 (Umetrics, Umea, Sweden) for the multivariate statistical analysis. Matlab (The MathWorks, Naticks, MA, USA) was also used, when needed, to correct intensity drop through sequence using the toolbox freely available online at https://github.com/Biospec/cluster‐toolbox‐v2.0.

### 2.8 Statistical Analysis

Data matrices obtained for tissue and EVs at positive and negative ionization modes were process with SIMCA‐P and Matlab to perform multivariate (MVA) and univariate (UVA) statistical analysis, respectively. MVA and UVA statistical analysis were carried out to determine differences between AD patients and CTRL individuals in both biological samples. Also, to determine if gender and/or sex should be considered as covariables, independent *t*‐tests were performed in PSPP (https://www.gnu.org/software/pspp/). For MVA, SIMCA‐P software was used for both unsupervised (PCA—Principal Component Analysis) and supervised analysis (PLS‐DA—Partial Least Squares Discriminant Analysis). PCA was used to reduce dimensionality and to study data quality, assess the reliability of the analytical procedure, visualize tendencies between groups and determine the presence of outliers. Samples out of Hotelling's T2 (95% confidence level) as well as QC samples were removed for further supervised analysis. Model quality was firstly analysed by the explained variance (R2) and the predicted variance (Q2). R2 and Q2 had to be greater than 0.6 and 0.4, respectively, or the difference between them was less than 0.3 (Godzien et al. 2013). Afterwards, PLS‐DA was performed to model the relationship between the measured features and the target class label (AD and CTRL), and it was followed by a suitable validation method by cross‐validated analysis of variance (CV‐ANOVA) (Godzien et al. 2013). PLS‐DA models that were validated were used for variable and subsequent feature selection. In this sense, a variable importance on projection (VIP) score and an absolute value of p(corr) greater than 1.0 and 0.5, respectively, were used as cutoff points for variable selection (Wheelock and Wheelock 2013).

For UVA, a non‐parametric test was applied. The U‐Mann Whitney test (*p* value < 0.05) was used to determine whether the feature was significant between AD patients and CTRL individuals. Then, Benjamini–Hochberg was applied to control the false discovery rate at a level of *α* = 0.05 (*q* value < 0.05) and log_2_ – false change (log2FC) was calculated as log_2_FC = log_2_ (average AD/average CTRL). The criteria used for variable selection in UVA was *q* value < 0.05 and log_2_FC greater than 1 or less than –1 that means more than the double abundance in one group in respect to the other.

### 2.9 Metabolic Feature Identification

The selected features in previous steps were identify based on the *m/z* of the metabolite and the fragmentation pattern obtained by MS/MS analysis followed the conditions described previously and comparing them with in‐house and online databases, as well as with commercial standards. To distinguish confidence levels in annotation accuracy, the metabolomics standards initiative (MSI) in 2007^47,^ (Fiehn et al. 2007) proposed four identification levels, which were later expanded to five levels (Schymanski et al. 2014). In this sense, all metabolites were properly annotated following MSI criteria. Features assigned to metabolites have to fulfill an appropriate mass accuracy (maximum error mass 10 ppm).

### 2.10 Metabolic Pathway Analysis

Identified metabolites were used for further pathway enrichment analysis and pathway topology analysis, both integrated in the metabolic pathway analysis available in Metaboanalyst 6.0 web‐based platform (https://dev.metaboanalyst.ca/MetaboAnalyst/home.xhtml). The type of pathway enrichment analysis used was the overrepresentation analysis (ORA). To calculate the *p* value the hypergeometric test, which evaluates whether compounds involved in a particular pathway are enriched compared to random hits, was used (Xia and Wishart 2011). The computed *p* value for each metabolic pathway indicates the probability of observing at least a particular number of metabolites from a certain metabolite set in a given compound list (Xia and Wishart 2011). For the pathway topology analysis, the relative‐betweenness centrality algorithm was used. This algorithm measures the number of shortest paths going through the node, and thus, it considers the global network structure not only the immediate neighbour of the current node. The quantitative measure of the position of a node relative to the other nodes (centrality measure) is a common metric used in graph theory to estimate the relative importance of individual nodes to the overall network (Xia and Wishart 2011; Xia, Wishart, and Valencia 2011). The pathway impact score, that was calculated as the sum of the importance measures of the matched metabolites normalised by the sum of the importance measures of all metabolites in each pathway, was also obtained and considered as cut‐off points for pathways selection (Xia, Wishart, and Valencia 2011). The selected pathway library as reference metabolic pathway was the homo sapiens from the Kyoto Encyclopedia of Genes and Genomes (KEGG) database. Metabolic pathway analysis based on enrichment and topology analysis procedures allowed the identification of the most relevant metabolic pathways via pathway impact and adjusted *p* value obtained using the False Discovery Rate (FDR) approach.

### 2.11 Brain Heterogeneity—Study and Comparison of Four Brain Regions

To analyse brain heterogeneity, 1 mg of tissue and bdEVs isolated from the four brain regions were prepared as described above and analysed following the same untargeted metabolomics approach to determine any metabolic differences associated with different brain regions as well as to follow up the distribution of the identified metabolites in both, tissue and bdEVs. For this purpose, all regions were obtained from one healthy control (male; 89‐years‐old) and one AD patient (male; 83‐years‐old) due to the difficulties of getting tissue from many individuals. Detailed information is gathered in Table S2.

## 3 Results

### 3.1 Brain Tissue Selection for Metabolomics Analysis

**FIGURE 1:**
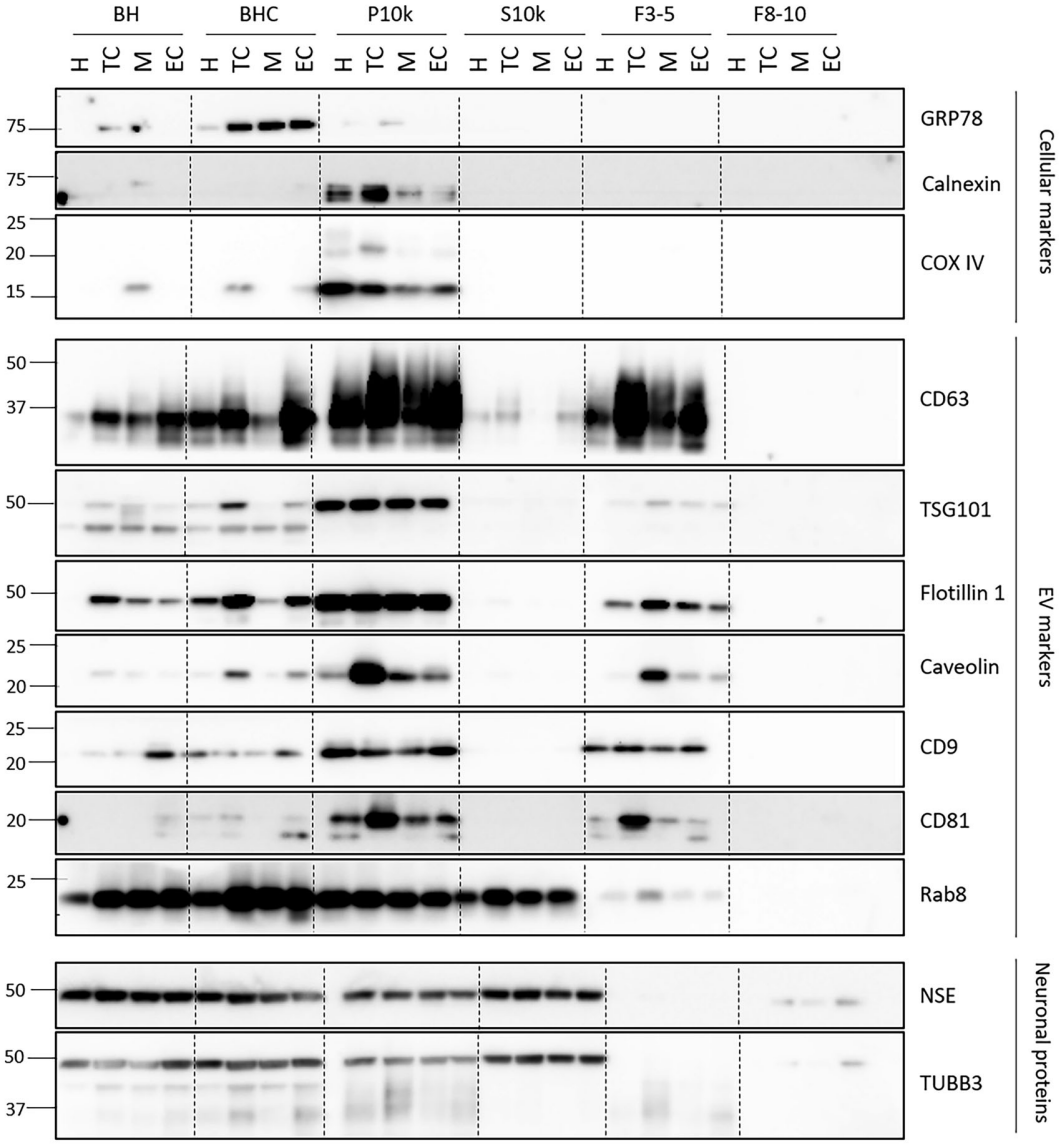
Western blotting analysis of samples from four different brain regions in AD patients. F3‐5 were enriched in EV markers (CD63, TSG101, Flotillin 1, Caveolin, CD9, CD81, Rab8) and void of cellular markers (GRP78, Calnexin, COX IV). Neuron‐specific enolase (NSE) was present in F8‐10, but not in F3‐5. BH, brain homogenate; BHC, brain homogenate after treatment with collagenase; P10k, pellet after 10,000 × *g* centrifugation; S10k, supernatant after 10,000 × *g* centrifugation; F3‐5, SEC‐fractions EV‐enriched; F8‐10, SEC‐fractions protein‐enriched. Brain regions: H = hippocampus, TC = temporal cortex, M = midbrain, EC = entorhinal cortex. Molecular weights are shown in kDa.

To apply successfully metabolomic analysis to bdEVs samples from AD patients and healthy controls, EVs were isolated from frozen human brain tissues by adapting a protocol established previously by Huang et al. (2020). To select the EVs’ enriched brain region for further analysis, the F3‐5 fractions pool from the EV preparation and the BH and BHC supernatants from the tissue homogenates were characterised by Western blotting. EV purity was evaluated by testing for cellular marker proteins such as GRP78, calnexin and COX IV. It was observed that the EV preparations were void of those contaminants (Figure 1), and they were enriched in EV marker**s (**CD63, TSG101, Flotillin 1, Caveolin, CD9, CD81 and Rab8). Furthermore, the neuron‐specific proteins enolase (NSE, a peripheral brain damage marker) and tubulin betaIII (TUBB3) were present in fractions F8‐10, but not in F3‐5. NSE is a soluble protein, primarily located in the cytoplasm of neurons and neuroendocrine cells, released into extracellular spaces during neuronal damage or stress (Haque et al. 2018). For that reason, NSE elutes in SEC‐fractions F8‐10, which are the protein‐enriched ones. As expected, the P10k, which is a less purified EV population, was positive for both EV and cellular markers. This indicates that the isolation protocol is optimal for the EV enrichment with minimal co‐isolation of cellular contaminants. The EV‐enriched fractions (F3‐5) showed higher expression of EV markers in the temporal cortex compared to other brain regions (Figure 1). Therefore, we selected the temporal cortex region for further bdEVs characterization as well as for untargeted metabolomics analysis. In this last case, a pool of fractions from 3 to 5 was prepared in order to extract metabolites.

### 3.2 Temporal Cortex‐derived EV Characterization

**FIGURE 2:**
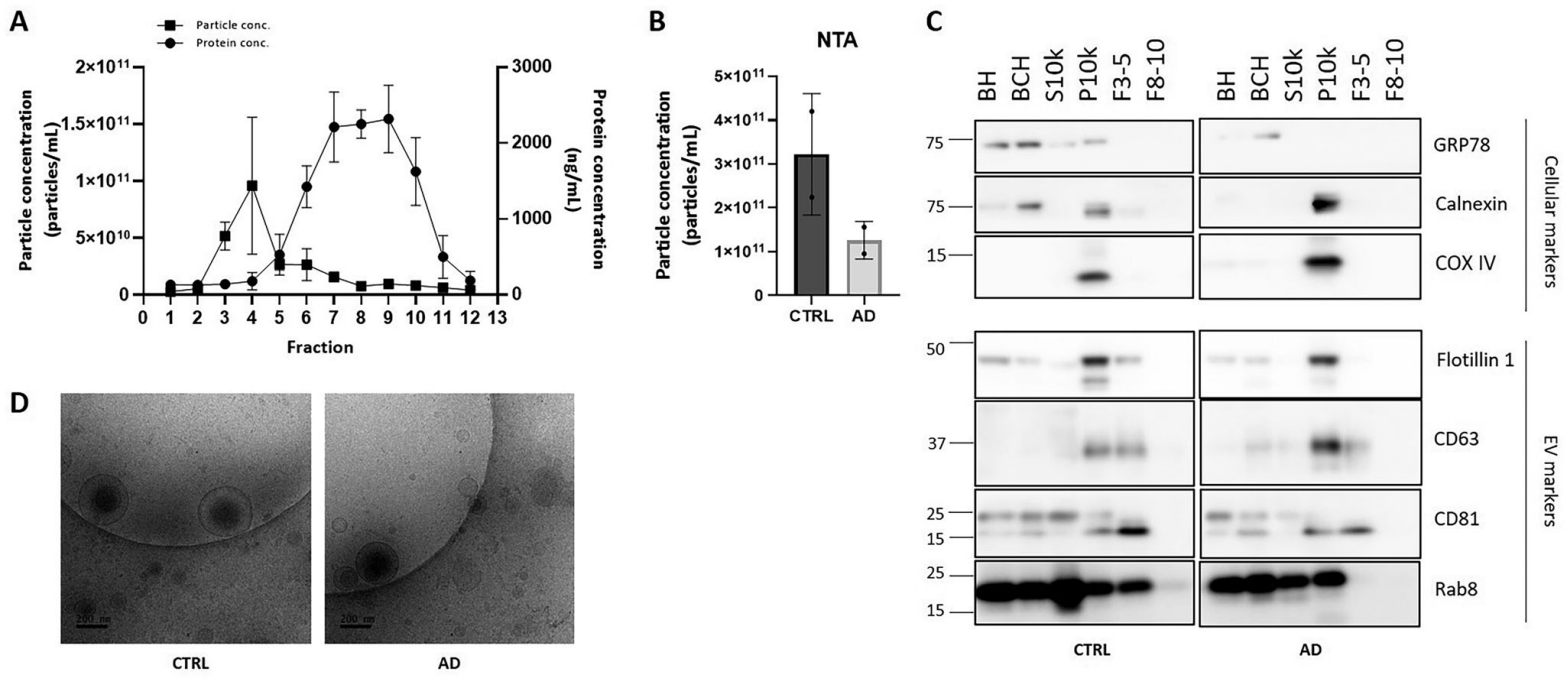
Characterization of EVs isolated from human temporal cortex by SEC. (A) Particle concentration (left y‐axis) and protein concentration (right y‐axis) of each fraction were measured by NTA and Bradford assay, respectively. The mean and standard deviation of three samples are shown (*n* = 3). (B) Particle concentration of EV preparations (F3‐5) was measured by NTA, comparing both AD and CTRL groups. The mean and standard deviation of two samples per group are shown (*n* = 2). U‐Mann Whitney test, *p* value 0.3333 (ns). (C) Representative Western blotting membranes from AD and CTRL samples. F3‐5 were enriched in EV markers (Flotillin 1, CD63, CD81, Rab8) and void of cellular markers (GRP78, Calnexin, COX IV). BH, brain homogenate; BHC, brain homogenate after treatment with collagenase; P10k, pellet after 10,000 × *g* centrifugation; S10k, supernatant after 10,000 × *g* centrifugation; F3‐5, SEC‐fractions EV‐enriched; F8‐10, SEC‐fractions protein‐enriched. Molecular weights are shown in kDa. (C) Representative images of cryo‐EM of EVs from AD and CTRL samples present in fraction 4. The scale bar represents 200 nm.

Since the temporal cortex was the brain tissue most highly EV‐enriched, SEC‐fractions from the temporal cortex were fully characterised by NTA, Bradford assay and Western blotting analysis to study particle and protein concentration, and protein‐content. NTA and Bradford assays showed that fractions F3, F4 and F5 presented a high content of particles (being F4 the one with the highest particle concentration), while fractions F8, F9 and F10 were protein‐enriched (Figure 2A). When comparing particle concentration between AD and control groups by NTA, we could see that particle number in the pool of Fractions 3–5 (EV preparations) is slightly lower in AD samples compared to control groups, with no significant differences (Figure 2B). Consistent with the previous results from Western blotting analysis, the EV preparations were positive for EV markers (Flotillin 1, CD63, CD81 and Rab8), and they were free of cellular debris and contaminants as they were void of markers as GRP78, Calnexin and COX IV (Figure 2C). NSE levels are often elevated in conditions of neurodegeneration, including AD. Higher NSE concentrations in F8‐10 in AD compared to controls could point to increased neuronal injury, axonal degradation or metabolic dysfunction specific to AD pathology (Haque et al. 2018). However, no differences in NSE expression between AD and controls have been seen in the temporal cortex tissue by WB analysis (Figure S2). These results were further confirmed by cryo‐EM in both AD and CTRL groups, where EVs visualised in fraction 4 (as representative of all EV‐enriched fractions) displayed a rounded morphology and sizes in a range of 50 and 250 nm. Moreover, there were no differences in the morphology of EVs between AD and control groups (Figure 2D).

### 3.3 UHPLC‐MS Metabolomics Analysis

Following the complete characterization of EVs, we performed the UHPLC‐MS metabolomic analysis with temporal cortex and EVs derived from this tissue of AD patients and controls (pool of F3‐5). To ensure adequate system reproducibility and stability through the analytical sequence, the system performance during acquisition, such as system pressure, base peak intensity (BPI), mass accuracy and overlapping of QCs, were first checked. Then, and before any statistical analysis, independent *t*‐test were performed in AD and CTRL groups to determine if gender and sex should be considered as co‐variables. In both cases, the significance for Levene's test for equality of variances was bigger than 0.05 (significance for gender = 0.132 and significance for age = 0.294), which means that equal variances could be assumed. The *t*‐test with 95% confidence *p* value was 0.349 for gender and 0.887 for age, which are non‐significant values. Thus, gender and age were not considered as co‐variables for further statistical analysis.

After the deconvolution process, alignment and matrix filtration, we detected 297 and 61 features for positive ionization mode for temporal‐cortex tissue and bdEVs samples, respectively, and 87 and 8 for negative ionization mode. Finally, normalization by median fold change was performed before any multivariate (MVA) or univariate statistical analysis (UVA). For detail information, see Table S3 for positive ionization mode and Table S4 for negative ionization mode.

**FIGURE 3:**
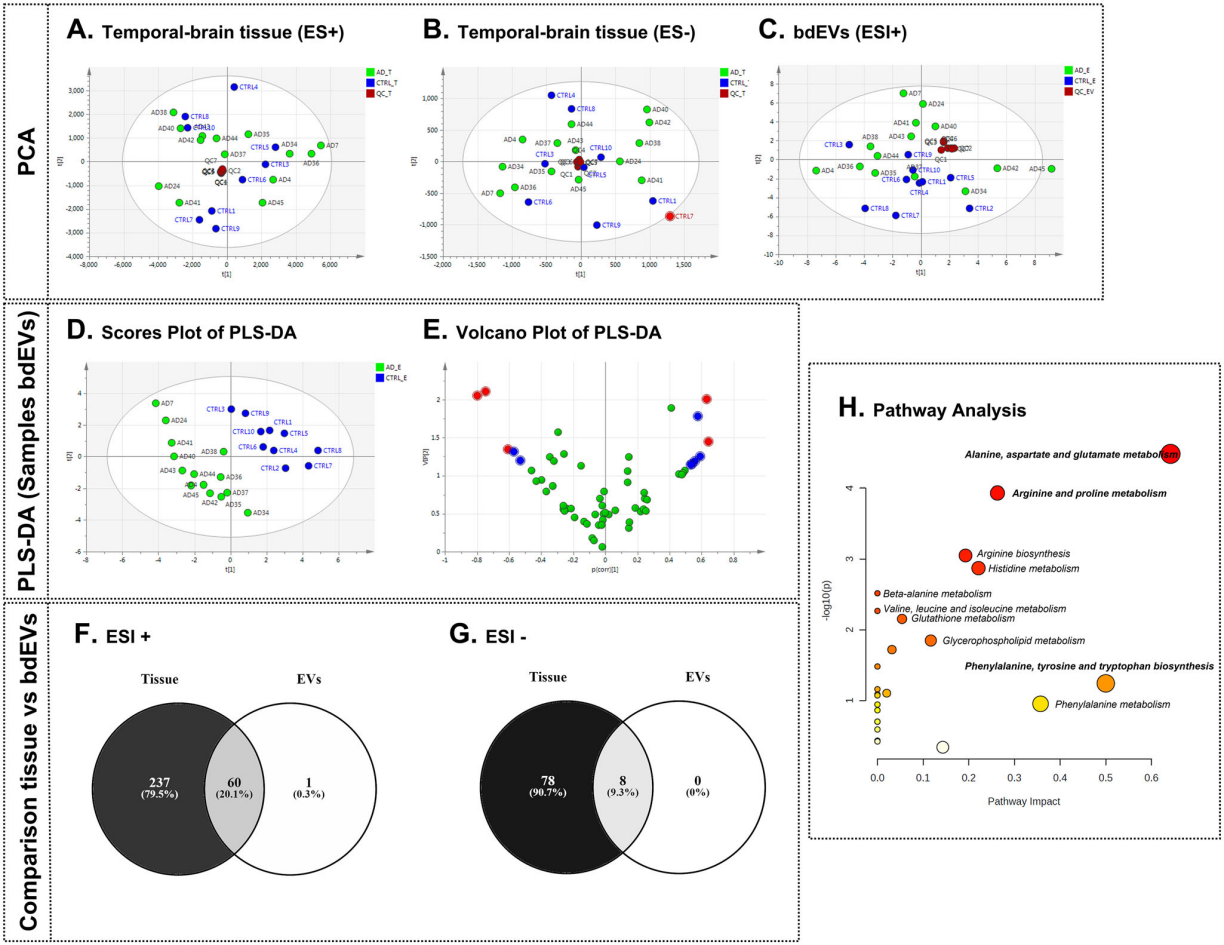
Untargeted metabolomics analysis of temporal‐brain tissue and bdEVs analysed at positive (ESI+) and negative (ESI–) ionization modes. (A) PCA scores plot of temporal‐brain tissue samples obtained at ESI+ (R2X = 0.486, Q2 = 0.216, 2 PCs and pareto scaling); (B) PCA scores plot of temporal‐brain tissue samples obtained at ESI‐ (R2X = 0.467, Q2 = 0.076, 2 PCs and pareto scaling). The red dot represents the outlier CTRL7; (C) PCA scores plot of bdEV samples obtained at ESI+ (R2X = 0.318, Q2 = 0.071, 2PCs, and pareto scaling); (D) PLS‐DA scores plot of bdEV samples obtained at ESI+ (R2X = 0.203, R2Y = 0.906, Q2 = 0.383, 2PCs, pareto scaling and CV‐ANOVA *p* value = 0.0298); (E) Volcano plot obtained from validated PLS‐DA model with selected features highlighted in blue (VIP > 1 and |0.5 < p(corr) > 0.6|) or red (VIP > 1 and |p(corr) > 0.6|); (F) Venn diagram of features obtained in temporal‐brain tissue and bdEVs at ESI+; (G) Venn diagram of features obtained in temporal‐brain tissue and bdEVs at ESI‐ and (H) Pathway analysis overview where higher pathway impact values represent the relative importance of the pathway, the size of the circle indicates the impact of the pathway and the color the significance (the more intense the red color, the lower the *p* value).

For MVA, first a PCA was done to reduce dimensionality and to find group clustering, tendencies within injection order and/or outliers in both biological matrices and ionization modes. A tendency in the QCs with the injection order at positive ionization mode (data not included) in both bdEVs and temporal‐cortex tissue samples was observed. Thus, a QC correction algorithm was used to avoid any time related effect. After this correction, a perfect clustering of QC samples was obtained (Figure 3A–C). The PCA of tissue at negative ionization mode, showed that sample CTRL7 was an outlier due to its location outside of the Hotelling's T2 ratio with 95% of confidence (Figure 3B). In the case of EVs, a distribution tendency was observed in the PCA, with AD samples located mainly in the upper part of PC2 whereas CTRL samples were in the lower part (Figure 3C). In the case of EVs at negative ionization mode, no PCA model was generated probably due to the scarce number of features obtained after data filtration (*n* = 8).

After the PCA, a supervised model based on PLS‐DA was built using AD or CTRL as labels. Only bdEVs at positive ionization mode generated a PLS‐DA model that fulfilled the validation criteria with a CV‐ANOVA *p* value of 0.0298 (see the scores plot in Figure 3D). Finally, 12 features from the PLS‐DA model that passed the criteria with VIP greater than 1 and absolute p(corr) greater than 0.5 (Figure 3E), influenced the separation of AD and control groups. After the identification process, Table 2 shows information related to each feature with the *m/z*, RT in minutes, metabolite name and confidence level based on the MSI (Sumner et al. 2007; Fiehn et al. 2007).

**TABLE 2:** Selected metabolites obtained from the PLS‐DA model that influence on AD and CTRL group separation.

Variable ID	RT	m/z	p(corr)	VIP	Tendency	Ionization Mode	Identification/Annotation	MSI
FTR1.37_134.0444	1.37	134.0444	0.6316	2.0057	↓	ESI+	NAA fragment	1
FTR1.37_158.0443	1.37	158.0443	0.5768	1.7802	↓	ESI+	NAA fragment	1
FTR1.37_130.0495	1.37	130.0495	0.6429	1.4459	↓	ESI+	NAA fragment	1
FTR7.97_613.1611	7.97	613.1611	0.5326	1.1434	↓	ESI+	Oxidised glutathione (GSSG)	1
FTR1.85_104.0704	1.85	104.0704	0.5580	1.1927	↓	ESI+	γ‐aminobutyric acid (GABA)	1
FTR2.02_154.0573	2.02	154.0573	0.5415	1.1511	↓	ESI+	Unknown	5
FTR2.03_132.0761	2.03	132.0761	0.5911	1.2527	↓	ESI+	Creatine	1
FTR1.73_132.1018	1.73	132.1018	−0.5712	1.3199	↑	ESI+	Isoleucine/Leucine	1
FTR1.67_188.0698	1.67	188.0698	−0.6085	1.3471	↑	ESI+	Tryptophan	1
FTR1.86_172.1437	1.86	172.1437	−0.5306	1.1959	↑	ESI+	Unknown	5
FTR1.67_132.1013	1.67	132.1013	−0.7985	2.0598	↑	ESI+	Isoleucine/Leucine	2
FTR1.72_166.0857	1.72	166.0857	−0.7490	2.1132	↑	ESI+	Phenylalanine	1

*Note*: Tendency: Referred to AD (i.e., up‐ or down‐regulated in AD compared to CTRL).

Abbreviations: MSI, metabolomics standard initiative; VIP, variable importance on projection.

To complement the statistical analysis with UVA, a Mann‐Whitney U test followed by Benjamini–Hochberg correction was applied and the fold change (log_2_(FC)) was calculated. In total, just one metabolite identified as phenylalanine had a significant *q* value (*q* value = 0.0193) in bdEVs, and 11 metabolites had more than double abundance in one group compared to the other (|log_2_(FC)|> 1) (Table S5).

Given the importance of the bdEVs’ biological role as vehicles in different diseases and the human body, we aimed to determine if the bdEVs’ metabolome reflects the metabolome of the tissue of origin. Therefore, a comparison study was performed between temporal‐cortex tissue and bdEVs isolated from that tissue samples. For this purpose, a Venn Diagram was built for positive and negative ionization modes (Figure 3F,G) and those common features (60 in positive and 8 in negative) were further identified (Table 3). In total 40 out of 60 and 6 out of 8 features were identified at different confidence levels (detailed information in Table S5). Among them, amino acids, carnitine and acylcarnitines, lysophosphatidylcholines, γ‐aminobutyric acid (GABA) were found (Table 3).

**TABLE 3:** Common features between brain tissue and EVs samples with the corresponding MSI confidence level and the ion identify for positive and/or negative modes.

Variable ID	RT (min)	*m/z*	Ionization mode	Identification/Annotation	Adduct	MSI
FTR1.04_524.3698	1.04	524.3698	ESI+	LPC 18:0	[M+H]^+^	2
FTR1.06_496.3393	1.06	496.3393	ESI+	LPC 16:0	[M+H]^+^	2
FTR1.06_518.3223	1.06	518.3223	ESI+	LPC 18:3	[M+H]^+^	2
FTR1.07_468.3077	1.07	468.3077	ESI+	LPC 14:0	[M+H]^+^	2
FTR1.16_232.1537	1.16	232.1537	ESI+	CAR 4:0	[M+H]^+^	2
FTR1.34_204.1229	1.34	204.1229	ESI+	CAR 2:0	[M+H]^+^	2
FTR1.36_371.0696	1.36	371.0696	ESI‐	NAA	[2M+Na‐H]^−^	1
FTR1.37_174.0402	1.37	174.0402	ESI‐	NAA	[M‐H]^−^	1
FTR1.45_248.1493	1.45	248.1493	ESI+	3‐Hydroxybutyrilcarnitine	[M+H]^+^	2
FTR1.50_327.0794	1.50	327.0794	ESI+	N‐Acetyl Aspartil Glutamic Acid (NAAG)	[M+H]^+^	1
FTR1.59_104.1067	1.59	104.1067	ESI+	Choline	[M+H]^+^	1
FTR1.66_137.0451	1.66	137.0451	ESI+	Hypoxanthine	[M+H]^+^	1
FTR1.67_114.0661	1.67	114.0661	ESI+	Creatinine	[M+H]^+^	1
FTR1.67_188.0698	1.67	188.0698	ESI+	Tryptophan	[M+H]^+^	1
FTR1.67_162.1124	1.67	162.1124	ESI+	L‐carnitine	[M+H]^+^	2
FTR1.67_132.1013	1.67	132.1013	ESI+	Isoleucine/Leucine	[M+H]^+^	2
FTR1.72_166.0857	1.72	166.0857	ESI+	Phenylalanine	[M+H]^+^	1
FTR1.73_132.1018	1.73	132.1018	ESI+	Isoleucine/Leucine	[M+H]^+^	2
FTR1.8_268.1046	1.80	268.1046	ESI+	Adenosine	[M+H]^+^	1
FTR1.85_104.0704	1.85	104.0704	ESI+	GABA	[M+H]^+^	2
FTR1.91_137.0449	1.91	137.0449	ESI+	Inosine	[M+H]^+^	1
FTR2.03_130.0625	2.03	130.0625	ESI‐	Creatine	[M+H]^+^	1
FTR2.03_132.0761	2.03	132.0761	ESI+	Creatine	[M+H]^+^	1
FTR2.07_144.1015	2.07	144.1015	ESI+	Proline‐Betaine	[M+H]^+^	4
FTR3.02_146.0463	3.02	146.0463	ESI‐	Glutamic acid	[M+H]^+^	1
FTR3.02_148.0599	3.02	148.0599	ESI+	Glutamic acid	[M+H]^+^	1
FTR3.39_134.044	3.39	134.0440	ESI+	Aspartic acid	[M+H]^+^	1
FTR3.65_147.0756	3.65	147.0756	ESI+	Glutamine	[M+H]^+^	1
FTR4.03_188.1751	4.03	188.1751	ESI+	N1‐Acetylspermidine or N8‐Acetylspermidine	[M+H]^+^	3
FTR4.32_258.1099	4.32	258.1099	ESI+	Glycerophosphocholine	[M+H]^+^	1
FTR4.82_216.063	4.82	216.0630	ESI+	Glytherophophoethanolamine	[M+H]^+^	3
FTR4.86_214.0488	4.86	214.0488	ESI‐	Glytherophophoethanolamine	[M+H]^−^	3
FTR6.11_175.1184	6.11	175.1184	ESI+	Arginine	[M+H]^+^	1
FTR6.32_156.0765	6.32	156.0765	ESI+	Histidine	[M+H]^+^	1
FTR6.51_147.1122	6.51	147.1122	ESI+	Lysine	[M+H]^+^	1
FTR7.63_223.075	7.63	223.0750	ESI+	Cystathione	[M+H]^+^	1
FTR7.97_613.1611	7.97	613.1611	ESI+	Oxidised glutathione (GSSG)	[M+H]^+^	1
FTR8.11_146.1647	8.11	146.1647	ESI+	Spermidine	[M+H]^+^	1

### 3.4 Metabolic Pathway Analysis

A compound list with all the identified metabolites commonly obtained in tissue and bdEVs (Figure 3F,G) was used to perform the pathway analysis based on enrichment and topology analysis in Metaboanalyst 6.0. According to the *p* value from the pathway enrichment analysis and the pathway impact values from the topology analysis, 24 pathways were identified (Figure 3H). The information with the total number of metabolites included in each pathway, the hits or metabolites observed in the input compound list, raw *p* value, adjusted *p* values, FDR and the pathway impact is presented in Table 4. The most relevant pathways were identified via the pathway impact and the adjusted *p* value, based this *p* value on the FDR for multiple testing corrections. As mentioned before, the pathway impact score was calculated as the sum of the importance measures of the matched metabolites normalised by the sum of the importance measures of all metabolites in each pathway, and it refers to the influence of a specific pathway on AD associated with the metabolites that are relevant for this condition. Taking into consideration both measurements, five pathways had FDR *p* value less than 0.05 (Figure 3H, Table 4), and one had a pathway impact of 0.5 (Figure 3H, Table 4). The alanine, aspartate and glutamate metabolism pathway had an FDR *p* value of 0.0026 and a pathway impact of 0.642, indicating that is the most relevant pathway in this study. Among the 28 total metabolites included in this pathway, five metabolites (NAA, aspartic acid, GABA and NAAG) were found in temporal‐cortex tissue as well as in bdEVs with the same tendency. Noteworthy, NAA and GABA were found to be significant in the bdEVs PLS‐DA model (Table 2). The identification of these five metabolites in bdEVs presents a special interest due to their known relevance in AD disease. Interestingly, NAA in bdEVs was found to significantly separate AD and CTRL individuals. Similarly, the arginine and proline metabolism pathway, the arginine biosynthesis and the histidine metabolism had FDR *p* values less than 0.05 and impact values less than 0.3 (Table 4). Notably, the phenylalanine, tyrosine and tryptophan biosynthesis pathway displayed a high impact of 0.5 (Table 4).

**TABLE 4:** Pathway analysis results obtained by metaboanalyst.

Pathway	Total	Expected	Hits	Raw *p* value	‐log10(p)	Holm adjust	FDR	Impact
**Alanine, aspartate, and glutamate metabolism**	**28**	**0.40889**	**5**	**3.3E‐05**	**4.4812**	**0.0026**	**0.0026**	**0.642**
Arginine and proline metabolism	36	0.52571	5	1.2E‐04	3.9309	0.0093	0.0047	0.263
Arginine biosynthesis	14	0.20444	3	8.9E‐04	3.0493	0.0696	0.0238	0.193
Histidine metabolism	16	0.23365	3	1.3E‐03	2.8705	0.1037	0.0269	0.221
beta‐Alanine metabolism	21	0.30667	3	3.1E‐03	2.5156	0.2318	0.0488	0.000
Valine, leucine, and isoleucine biosynthesis	8	0.11683	2	5.4E‐03	2.2662	0.4063	0.0722	0.000
Glutathione metabolism	28	0.40889	3	7.0E‐03	2.1532	0.5200	0.0803	0.054
Glycerophospholipid metabolism	36	0.52571	3	1.4E‐02	1.848	1.0000	0.1419	0.117
Butanoate metabolism	15	0.21905	2	1.9E‐02	1.7192	1.0000	0.1697	0.032
Ether lipid metabolism	20	0.29206	2	3.3E‐02	1.4809	1.0000	0.2644	0.000
**Phenylalanine, tyrosine, and tryptophan biosynthesis**	**4**	**0.058413**	**1**	**5.7E‐02**	**1.2426**	**1.0000**	**0.4160**	**0.500**
Lysine degradation	30	0.4381	2	6.9E‐02	1.1594	1.0000	0.4513	0.000
Purine metabolism	70	1.0222	3	7.9E‐02	1.1036	1.0000	0.4513	0.020
Glycine, serine, and threonine metabolism	33	0.4819	2	8.2E‐02	1.0866	1.0000	0.4513	0.000
Nitrogen metabolism	6	0.087619	1	8.5E‐02	1.0726	1.0000	0.4513	0.000
Phenylalanine metabolism	8	0.11683	1	1.1E‐01	0.95366	1.0000	0.5358	0.357
Valine, leucine, and isoleucine degradation	40	0.58413	2	1.1E‐01	0.94367	1.0000	0.5358	0.000
Biotin metabolism	10	0.14603	1	1.4E‐01	0.86275	1.0000	0.6096	0.000
D‐Amino acid metabolism	15	0.21905	1	2.0E‐01	0.70156	1.0000	0.7952	0.000
Nicotinate and nicotinamide metabolism	15	0.21905	1	2.0E‐01	0.70156	1.0000	0.7952	0.000
Pantothenate and CoA biosynthesis	20	0.29206	1	2.6E‐01	0.59137	1.0000	0.9761	0.000
Porphyrin metabolism	31	0.4527	1	3.7E‐01	0.43296	1.0000	1.0000	0.000
Glyoxylate and dicarboxylate metabolism	32	0.4673	1	3.8E‐01	0.42204	1.0000	1.0000	0.000
Tryptophan metabolism	41	0.59873	1	4.6E‐01	0.3399	1.0000	1.0000	0.143

### 3.5 Brain Heterogeneity—Study of Four Brain Regions

**FIGURE 4:**
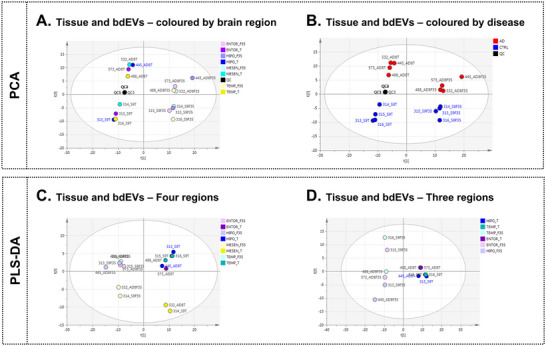
Untargeted metabolomics analysis of brain tissue and bdEVs isolated samples obtained from the four brain regions at positive (ESI+) ionization mode. (A) PCA scores plot colored based on brain region (ENTOR = entorhinal cortex, HIPO = hippocampus, MESEN = midbrain, and TEMP = temporal cortex) (_T = tissue, _F35 = bdEV) (R2X = 0.776, Q2 = 0.441, 4 PCs and autoscaling); (B) PCA scores plot colored based on disease (AD = Alzheimer's disease patient, CTRL = healthy control individual, and QC = quality control samples); (C) PLS‐DA scores plot containing the four‐brain region (R2X = 0.505, R2Y = 0.275, Q2 = 0.0631, 2PCs, autoscaling and CV‐ANOVA *p* value = 0.99), and (B) scores plot containing only three regions (R2X = 0.610, R2Y = 0.314, Q2 = –0.028). ENTOR, entorhinal cortex; HIPO, hippocampus; MESEN, midbrain; TEMP, temporal cortex; _T, tissue, _F35, bdEV.

Bearing in mind the brain heterogeneity, analysis of four different brain regions, including the previous temporal cortex and adding midbrain, hippocampus and entorhinal cortex, was performed. Following the same workflow mentioned in previous sections, we built an unsupervised PCA with both tissue and bdEVs biological matrices to determine sample distribution and to assess analytical performance. Samples were coloured based on regions (Figure 4A) or based on disease (AD and CTRL) (Figure 4B), and a separation tendency in PC2 due to disease, and in PC1 due to biological matrix (bdEVs or tissue) was observed. This is not of special significance since only one AD patient and one healthy individual were used for this approximation. However, after performing a PLS‐DA using each region as label for the supervised model (Figure 4C), we observed that the bdEVs were related with the region used for the isolation. As an example, midbrain tissue and bdEVs isolated from midbrain were located in the lower part of the scores PLS‐DA plot. This indicates that the polar fraction of the metabolome in the midbrain is different from the other regions. Doing the same but removing midbrain samples and forcing model generation to enhance the separation of the other three regions to analyse the behaviour, we observed a slight tendency of separation between the hippocampus and the remaining regions (Figure 4D). These results were of special relevance not only to confirm the heterogeneity of the brain but also to confirm that EVs were related to the brain region where they were isolated, indicating the specificity based on sample type. These findings should be further studied due to the poor quality of PLS‐DA models that would be due to the scarce number of replicates. Finally, each feature was studied independently to verify that the midbrain and hippocampus were the most distinct regions. We found that the hippocampus and midbrain were the regions that contained the highest signals for most of the features.

## 4 Discussion

AD is a multifactorial neurodegenerative disease characterised by progressive cognitive decline and behavioural changes that constitute a worldwide public health problem. The biological mechanisms of AD onset and progression are not completely understood. In this study, we implemented metabolomics of bdEVs to investigate the main biochemical pathways altered in AD. EVs metabolomics is particularly useful and has huge potential in the research of AD pathology (Palomo et al. 2014; Wu et al. 2022). First, diverse metabolic changes have been associated with AD. Therefore, metabolomics provides a powerful approach to identify and explore new hallmarks of AD (Paglia et al. 2016; Kim et al. 2019; Batra et al. 2023; Horgusluoglu et al. 2022). Second, the application of EV metabolomics to AD is a worthy and viable approach to improve the diagnosis and treatment, since EVs can cross physiological barriers, circulate in body fluids, carry bioactive molecules, and are drug delivery agents for personalised medicine (Gomes et al. 2022; Elliott and He 2021). In fact, the value of EV metabolomics has been shown as well in other diseases such as liver disease and cancers of the prostate and pancreas (Williams et al. 2019; Clos‐Garcia et al. 2018; Puhka et al. 2017; Royo et al. 2017). We have specifically chosen human postmortem brain tissue from clinically characterised AD patients and healthy controls, as it is the most valuable tissue for the identification of new pathological reactions linked to AD. We obtained a detectable and quantifiable number of metabolites from the bdEVs samples by adapting a protocol for isolating EVs from the brain (Huang et al. 2020; Vella et al. 2017; Cheng et al. 2020). Our successfully improved isolation method showed EVs enrichment in the temporal cortex. The characterisation of the bdEVs preparations based on the International Society for Extracellular Vesicles requirement guidelines, showed the enrichment of EVs positive markers and the lack of cellular debris and contaminants (Théry et al. 2018; Welsh et al. 2024).

To discover AD‐associated specific metabolites, we performed an untargeted metabolomic approach with two different ionisation modes (ESI+ and ESI–) using temporal cortex and EVs derived from this tissue from AD patients and healthy controls. We detected 384 features for the temporal cortex (297 for positive and 87 for negative ionization mode) and 69 features for bdEVs (61 for positive and 8 for negative ionization mode) with 68 (60 for positive and 8 for negative ionization mode) common features. Interestingly, the metabolomic profiles of EVs showed a clear separation between AD patients and controls, with 12 relevant features obtained by the validated PLS‐DA model. These results confirm the feasibility of our isolation method to identify metabolites in bdEVs and show their powerful application to study AD.

Our metabolic pathway enrichment analysis with all the identified metabolites showed that the two most relevant pathways with the highest impact were the phenylalanine, tyrosine and tryptophan biosynthesis (impact 0.5) and the alanine, aspartate and glutamate pathway (impact 0.642). We showed that phenylalanine was significantly upregulated, and tryptophan was increased in bdEVs from the AD temporal cortex compared to controls. Moreover, we found upregulation of phenylalanine and high levels of tyrosine in the temporal cortex of AD patients compared to controls. Therefore, our study confirms that the phenylalanine, tyrosine and tryptophan biosynthesis pathway is one of the metabolic pathways with high impact in AD. That means that this biosynthesis pathway could be significantly functionally affected in AD, and could be contributing to the disease's pathology, perhaps by affecting neurotransmitter levels, brain metabolism or other related processes. These aromatic aminoacids (AA) have important functions in brain physiology as they are precursors of the neurotransmitters serotonin and catecholamines (dopamine, norepinephrine and epinephrine) (Fernstrom and Fernstrom 2007). In line with our observations, upregulation of the phenylalanine metabolism has been reported previously in the hippocampus, entorhinal cortex and middle‐temporal gyrus of AD patients (Liu et al. 2021; Xu et al. 2016). Furthermore, the increase has been corroborated in studies with AD rat models (Nilsen et al. 2014). However, other studies using CSF and plasma samples have described its downregulation associated with the disease (Czech et al. 2012; González‐Domínguez, García‐Barrera, and Gómez‐Ariza 2015; Aquilani et al. 2022). Importantly, the reduction in CSF phenylalanine linked to AD was independent of the patient nutritional state (Aquilani et al. 2022). Whether these contradictory results are due to the different origin of the samples (brain vs. plasma or different brain regions), different stages of the disease or the use of different methodologies, is something that requires further research. The accumulation of phenylalanine in the brain indicates an inability to metabolize this amino acid, whereas reduced levels could indicate an increased consumption of Aas. Nevertheless, both situations of altered AA metabolism have fatal consequences for the brain. In AD, higher serum phenylalanine levels have been related to systemic immune activation (Wissmann et al. 2013). The high levels of phenylalanine in bdEVs could also increase inflammation in the brain by enhancing the immune response and inducing the release of pro‐inflammatory cytokines (Wyse et al. 2021). Another consequence of the high levels of bdEVs phenylalanine could be synaptic dysfunction as it has been shown that increased concentrations of phenylalanine are able to reduce the synaptic density as shown in mixed cortical cultures from mice (Hörster et al. 2006). In line with these observations, high levels of phenylalanine are found in the deficiency of the phenylalanine hydroxylase (PHA) enzyme, which converts phenylalanine to tyrosine. Interestingly, these high levels cause a decrease in serotonin, dopamine and noradrenaline synthesis in the brain (Curtius et al. 1981; Pietz et al. 1999; González et al. 2016), something that could also occur in AD with high levels of phenylalanine as identified in bdEVs in our study. On the other hand, low levels of phenylalanine in AD result in deficits in neurotransmission, that contribute to synaptic dysfunction and loss of synapsis and neurons (Liu et al. 2014). Moreover, the increase levels of tryptophan in bdEVs could be led to the deviation of its metabolic fate to the kynurenine pathway. This could cause oxidative damage to the brain as described for some of the metabolic intermediates in this pathway (Santamaría et al. 2001). Interestingly, a recent publication describes that a shift in tryptophan towards the kynurenine pathway is a component in AD (Fernandes et al. 2023).

Furthermore, and more important, we identified NAA and GABA metabolites being significant in the bdEVs PLS‐DA model. NAA, the most abundant amino acid in the brain synthesised in the mitochondria from aspartic acid and acetyl‐coenzyme A (Tallan, Moore, and Stein 1956), can suppresses the aggregation of Aβ42 peptide and other proteins (Warepam et al. 2021; Dollé et al. 2018). Thus, it is particularly interesting that NAA was significant in the separation of AD patients from controls. We observed similar downregulated tendencies of NAA, NAAG, aspartic acid, GABA and glutamic acid in the temporal cortex and in EVs from this tissue in AD patients. These five metabolites verify the importance of the alanine, aspartate and glutamate pathway in AD, which has the highest significance and impact factor in our enrichment study. The reduction of these metabolites in bdEVs and in the temporal cortex may have a negative impact on the energy source for AD neurons and on the glutamatergic neurotransmission. The most abundant amino acids in the brain involved in the energy supplies are glutamate and glutamine. The exchange of glutamate, GABA and glutamine between neurons and astrocytes is known as the glutamate/GABA‐glutamine cycle (Bak, Schousboe, and Waagepetersen 2006; Hertz 2013). This cycle links cellular metabolism and neurotransmitter recycling, being the astrocytes responsible not only for the metabolic support to neurons but also for supplying them with the mediator of glutamatergic neurotransmission. In AD, several components of the glutamate/GABA‐glutamine cycle are perturbed producing hyperactive glutamatergic signalling (Masliah et al. 1996; Smith et al. 1991), which could contribute to synaptic dysfunction and neurodegeneration (Andersen, Schousboe, and Verkhratsky 2022). However, the reduced levels that we observe in the temporal cortex and bdEVs of metabolites of this pathway could also impair the required neurotransmitter recycling and lead to synaptic abnormalities. Other metabolomic studies correlate this pathway with AD status using postmortem temporal and frontal cortex, which shows for example reduced levels of NAA (Kim et al. 2019; Botosoa et al. 2012; Zhang et al. 2019). However, in other studies, despite showing alterations in this pathway, no changes or accumulation of NAA have been associated with the disease (Paglia et al. 2016; Graham et al. 2014). Moreover, the neuropeptide NAAG which modulates glutamate signalling, is as well significantly reduced in AD patients (Jaarsma, Veenma‐Van Der Duin, and Korf 1994). Since mitochondria abnormalities in the brain of AD patients are a prominent feature in the pathogenesis of the disease (Reiss et al. 2024; Ashleigh, Swerdlow, and Beal 2023), the reductions in NAA concentration described in AD patients may represent mitochondrial dysfunction (Jaarsma, Veenma‐Van Der Duin, and Korf 1994). Furthermore, we show as well reduced levels of GABA in bdEVs from the temporal cortex, in agreement with other studies in which GABA and glutamate levels are significantly decreased in the temporal, medial frontal and parietal cortex of AD patients and CSF samples (Gueli and Taibi 2013; Bai et al. 2015; Bareggi et al. 1982; Zimmer et al. 1984). GABA is an important inhibitory neurotransmitter that helps to maintain normal neural circuitry and neural network activities. The loss and reduced activity of GABA inhibitory interneurons produce neuron over‐activity that finally affects the cognitive abilities of AD patients and mouse models (Verret et al. 2012; Fu et al. 2023). Because the GABA inhibitory interneurons are emerging as an attractive therapeutic target for the treatment of AD (Xu et al. 2020), the identification of GABA in bdEVs in this study strengthened their important roles in the treatment of the disease.

The progression of AD described by the abnormal Tau deposition initiates at the entorhinal cortex and midbrain and propagates to the hippocampus, temporal cortex and the rest of the neocortex (Jové et al. 2021; Braak et al. 2006). The preclinical phase in AD between 20 and 30 years before the diagnosis of dementia (Perl 2010; Bateman et al. 2012; Hof and Morrison 1996), offers an opportunity to prevent the progression of the disease. Here, we show that the metabolome profile of different regions of the brain is distinct, which is important for the diagnosis of the disease progression. This is on agreement with a recent metabolomic signature showing brain region specific neurodegeneration progression (Ambeskovic et al. 2023). Specifically, we detect the highest signals in the metabolome of the hippocampus and midbrain compared to other regions. Noteworthy, the metabolites found with bdEVs samples were related to the tissue of origin. Since bdEVs are well known non‐invasive biomarkers and have translational therapeutic potential, it will be interesting for future studies to determine if the metabolic features described in EVs from temporal cortex can be also identified in EVs derived from plasma of AD patients. Overall, we demonstrate in this study that untargeted metabolomics of bdEVs is a useful tool to detect altered metabolites associated with AD.

## Author Contributions

**Patricia Hernandez**: Conceptualization (equal), formal analysis (equal), methodology (equal), Writing–original draft (equal), writing–review and editing (equal)**. Elisabeth Rackles**: Conceptualization (equal), formal analysis (equal), funding acquisition (equal), investigation (equal), methodology (equal), writing–review and editing (equal). **Oihane E. Alboniga**: Conceptualization (equal), data curation (equal), formal analysis (equal), methodology (equal), supervision (equal), writing–original draft (equal), writing–review and editing (equal). **Pablo Martínez‐Lage**: Methodology (supporting). **Emma N. Camacho**: Resources (equal). **Arantza Onaindia**: Resources (equal). **Manuel Fernandez**: Resources (equal). **Ana Talamillo**: Conceptualization (equal), formal analysis (equal), writing–original draft (equal), writing–review and editing (equal). **Juan M. Falcon‐Perez**: Conceptualization (equal), funding acquisition (equal), project administration (equal), writing–review and editing (equal).

## Conflicts of Interest

The authors declare no conflicts of interest.

## Supporting information



Supporting Information

Supporting Information

Supporting Information

Supporting Information

Supporting Information

Supporting Information

Supporting Information

Supporting Information

## Data Availability

The data that support the findings of this study are available on request from the corresponding author. The data are not publicly available due to privacy or ethical restrictions.
