# Supplementary material for: Metabolic Profiling of Brain Tissue and Brain‐Derived Extracellular Vesicles in Alzheimer's Disease
Source: J Extracell Vesicles. 2025 Feb 3;14(2):e70043. doi: 10.1002/jev2.70043 (PMC11791017; doi:10.1002/jev2.70043)
Supplement: Supplementary file 1 — Supporting Information [file JEV2-14-e70043-s003.docx]

*Supplementary material*


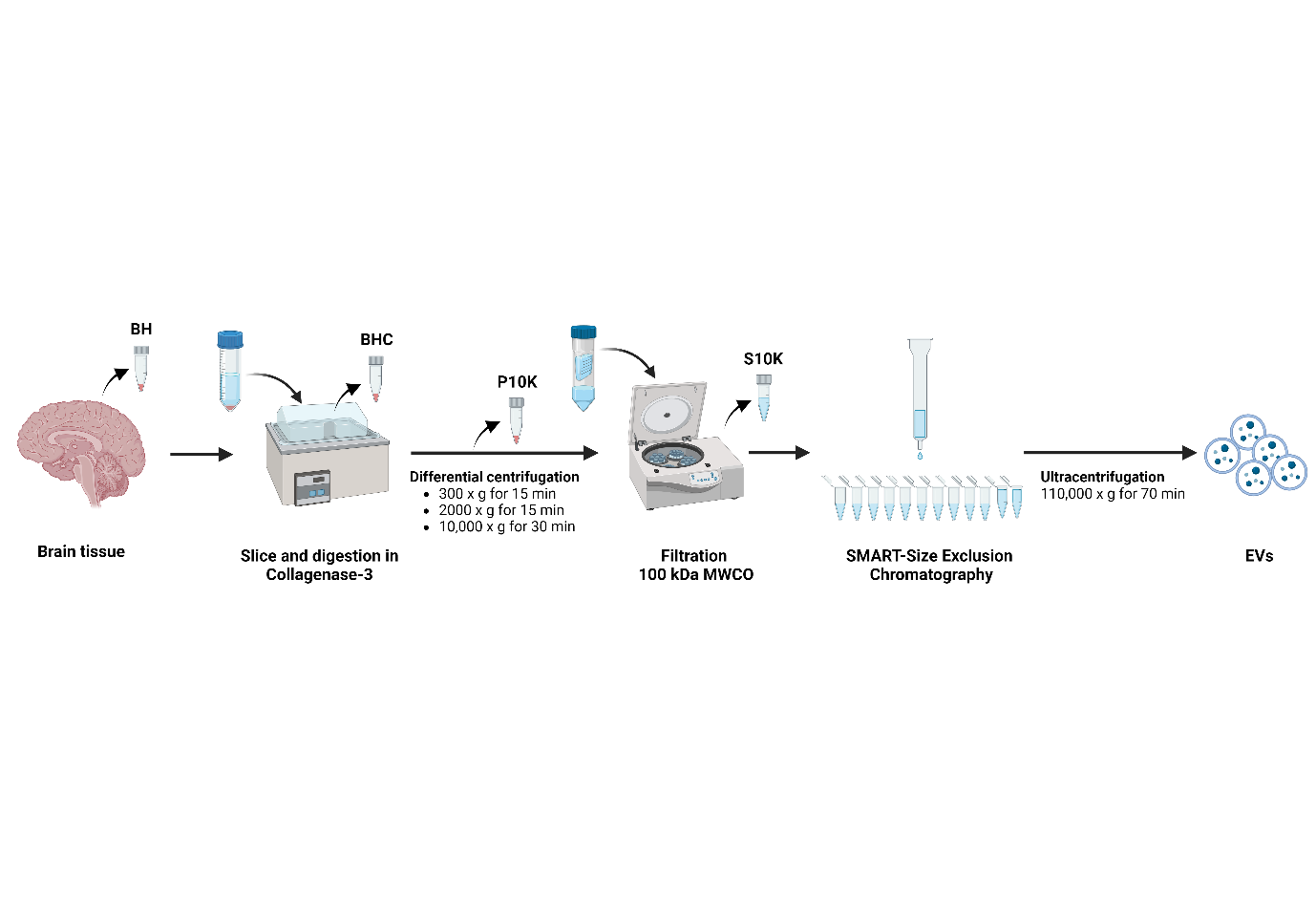


**FIGURE S1. Schematic of the protocol for isolation of EVs from human brain tissue.**
