# Supplementary material for: Metabolic Profiling of Brain Tissue and Brain‐Derived Extracellular Vesicles in Alzheimer's Disease
Source: J Extracell Vesicles. 2025 Feb 3;14(2):e70043. doi: 10.1002/jev2.70043 (PMC11791017; doi:10.1002/jev2.70043)
Supplement: Supplementary file 2 — Supporting Information [file JEV2-14-e70043-s005.docx]

*Supplementary material*

**TABLE S1. Clinical pathological information of the cohort analyzed in the study. AD: Alzheimer’s Disease, CTRL: healthy control. ABC classification of Alzheimer’s disease: A, ThaI Phase for β-amyloid plaques; B, Braak stage of neurofibrillary tangles; C, CERAD neuritic plaque score.**

| Case | Clinical diagnosis |
| --- | --- |
| AD2 | Alzheimer's disease variant with Lewy bodies. Moderate amyloid angiopathy. Moderate chronic vascular leukoencephalopathy. |
| AD4 | Alzheimer's disease Braak stage IV, CERAD stage C for β-amyloid. Amyloidotic angiopathy. Argirophilic grain disease stage IV. Ischemic-hemorrhagic lesions, with hyaline thrombi in small and medium caliber vessels affecting the right temporo-occipital lobe (6cm), left parietal lobe (4cm), right thalamus and striatum (5cm), vermis (5cm) with associated subarachnoid hemorrhage. Chronic vascular encephalopathy. |
| AD7 | Alzheimer's Dementia: Braak & Braak stage VI. CERAD stage C and Thal phase 3-4. Amyloid angiopathy in intraparenchymal vessels. Limbic subtype Lewy body disease (amygdala). |
| AD24 | Spinocerebellar atrophy. Limbic Lewy body disease (transitional). Neurodegenerative changes of Alzheimer-type disease (A1B3C1). Amyloid angiopathy type 1 (capillary). Cribriform lenticular nucleus. |
| AD34 | Alzheimer's disease (A1B3C2/3). Capillary and non-capillary amyloid angiopathy. Severe lenticular siderocalcinosis. |
| AD35 | Status spongiosus. Alzheimer's disease (A1B2C1/2). Non-capillary amyloid angiopathy. Hippocampal sclerosis. Severe hippocampal and lenticular siderocalcinosis. Cribriform lenticular nucleus. |
| AD36 | Alzheimer's disease (A3B3C2/3), frontal variant, with co-pathology of Lewy body disease of mesencephalic predominance, with limbic/transitional extension. Ischemia in the occipital white matter, 10mm maximum diameter. Non-capillary amyloid angiopathy. Severe lenticular siderocalcinosis. Cribriform lenticular nucleus. TX0090, D85430, M52200, M55100, M55400. |
| AD37 | Status spongiosus. Alzheimer’s disease (A1B3C2) with accompanying TDP43 and alpha-synuclein deposition. Hippocampal sclerosis. Capillary and non-capillary amyloid angiopathy. Arteriolosclerosis. |
| AD38 | Neurodegenerative changes of Alzheimer-type disease (A1B3C2). Changes suggestive of upper motor neuron disease. Vascular changes: Severe hippocampal, lenticular, cerebellar dentate nucleus, and cerebellar white matter siderocalcinosis. Cribriform lenticular nucleus and frontal white matter. No evidence of malignancy in the examined material. |
| AD40 | Intense decrease in the density of fine axons in the anterior roots, compared to the posterior roots. Decreased myelination of posterior columns, anterior horns, and lateral corticospinal tract, mainly at the cervical and thoracic levels, without a marked decrease in neuronal population. Neurodegenerative changes of Alzheimer-type disease (A1B2C1). |
| AD41 | Neurodegenerative changes of Alzheimer-type disease (A1B3C2). Subacute ischemia in the anterior hippocampus and middle and superior temporal cortex. Arteriolosclerosis. |
| AD42 | Leptomeningeal and parenchymal non-capillary amyloid angiopathy. Neurodegenerative changes of Alzheimer-type disease (A3B2C2/3).Ischemic-like changes in the frontal white matter. Moderate to severe lenticular, hippocampal, and internal capsule siderocalcinosis. Arteriosclerosis. Arteriolosclerosis. No evidence of malignancy in the examined material. |
| AD43 | Alzheimer's disease (A3B3C1) with associated α-synuclein deposition in the amygdala. Non-capillary amyloid angiopathy. Hippocampal sclerosis. Left superior parietal subdural lipoma, 2x1.5cm. |
| AD44 | Changes compatible with Alzheimer’s disease (A1B3C2). Chronic cerebellar infarct, 9mm maximum diameter. Focal and patchy amyloid deposits in leptomeningeal vessels. Cribriform lenticular nucleus. |
| AD45 | Degenerative changes of Alzheimer-type disease (A1/2B2C2). Amyloid angiopathy type II. Arteriosclerosis, arteriolosclerosis, and siderocalcinosis. |
| CTRL1 | Brain without significant lesions. |
| CTRL2 | Brain without remarkable histological alterations. |
| CTRL3 | No primary neurodegenerative changes. Subtle morphological and immunohistochemical alterations in the neocortex. |
| CTRL4 | Acute brainstem infarction located in the pons. Adjacent areas with signs of irreversible acute ischemia, including the cerebellum. No signs of neurodegeneration. |
| CTRL5 | No signs of primary neurodegeneration. Histologically normal brain. |
| CTRL6 | No signs of primary neurodegeneration. Normal aging. |
| CTRL7 | Hepatic encephalopathy. No signs of primary neurodegeneration. |
| CTRL8 | No evidence of primary neurodegenerative changes. |
| CTRL9 | No significant histological alterations. |
| CTRL10 | Mild arteriolosclerosis. No signs of primary neurodegeneration. |
