# Supplementary material for: Metabolic Profiling of Brain Tissue and Brain‐Derived Extracellular Vesicles in Alzheimer's Disease
Source: J Extracell Vesicles. 2025 Feb 3;14(2):e70043. doi: 10.1002/jev2.70043 (PMC11791017; doi:10.1002/jev2.70043)
Supplement: Supplementary file 3 — Supporting Information [file JEV2-14-e70043-s002.docx]

*Supplementary material*

**FIGURE S2.** Characterization of EVs isolated from human temporal cortex by SEC. Representative Western blotting membranes from AD and CTRL samples. NSE is present in soluble SEC-fractions, not in F3-5. BH **=** brain homogenate**,** BHC = brain homogenate after treatment with collagenase, P2k = pellet after 2,000 x g centrifugation, P10k = pellet after 10,000 x g centrifugation, F1-12 = SEC-fractions. Molecular weights are shown in kDa.
