# Supplementary material for: Metabolic Profiling of Brain Tissue and Brain‐Derived Extracellular Vesicles in Alzheimer's Disease
Source: J Extracell Vesicles. 2025 Feb 3;14(2):e70043. doi: 10.1002/jev2.70043 (PMC11791017; doi:10.1002/jev2.70043)
Supplement: Supplementary file 4 — Supporting Information [file JEV2-14-e70043-s007.docx]

*Supplementary material*

**TABLE S2. Sample information of individuals used to study brain heterogeneity. AD: Alzheimer’s Disease, CTRL: healthy control; M: male; F: female, and PMI: post-mortem interval.**

| Case | Age | Sex | Braak stage | PMI (h) | Tissue | Weight (mg) |
| --- | --- | --- | --- | --- | --- | --- |
| AD8 | 83 | M | IV | 18 | Hippocampus | 58.8 |
|  |  |  |  |  | Temporal cortex | 64.0 |
|  |  |  |  |  | Midbrain | 53.3 |
|  |  |  |  |  | Entorhinal cortex | 49.7 |
| CTRL9 | 89 | M | - | 3 | Hippocampus | 51.5 |
|  |  |  |  |  | Temporal cortex | 57.8 |
|  |  |  |  |  | Midbrain | 56.2 |
|  |  |  |  |  | Entorhinal cortex | 53.5 |
