# Supplementary material for: Metabolic Profiling of Brain Tissue and Brain‐Derived Extracellular Vesicles in Alzheimer's Disease
Source: J Extracell Vesicles. 2025 Feb 3;14(2):e70043. doi: 10.1002/jev2.70043 (PMC11791017; doi:10.1002/jev2.70043)
Supplement: Supplementary file 8 — Supporting Information [file JEV2-14-e70043-s004.docx]

*Supplementary material*

**Reagent and solutions**

The reagents and solutions used for EVs isolation were: Hibernate-E medium (Thermo Fisher, #A12476-01), collagenase type 3 (Worthington #CLS-3, #S8P18814), PhosSTOP phosphatase inhibitor (Sigma-Aldrich, #4906837001), complete protease inhibitor (Sigma-Aldrich, #11697498001), and Dulbecco’s phosphate buffered saline (DPBS; Gibco, #14190250). For brain tissue homogenization and protein quantification zirconium oxide beads (Bertin Technologies, #P000927-LYSKO-A.0) and Bradford protein assay (Bio-Rad, #5000006) were used.

For metabolites extraction and untargeted metabolomics analysis, LC-MS grade formic acid, acetonitrile (ACN), and water (H_2_O) were used for the mobile phases’ preparation and were purchased from Fisher Scientific (Pittsburgh, PA, USA). The methanol (MeOH) and chloroform used in metabolite extraction during sample preparation were obtained from Fisher Scientific (Pittsburgh, PA, USA) and Sigma-Aldrich (Steinheim, Germany), respectively. Standard reagents, which were methionine (13CD3), 5’-methylthioadenosine (MTA), choline, betaine, S-adenosyl homocysteine (SAH), S-adenosyl methionine (SAMe), spermidine and spermine, used to assess the proper LC-MS system operation were also purchased form Sigma-Aldrich (Steinheim, Germany) and prepared at a final concentration of 10 µM in ACN/H_2_O (60/40, v/v).

For western blotting to characterize bdEVs, LDS Sample Buffer, 1X (Invitrogen, #NP0008), NuPAGE 4–12% Bis-Tris pre-casted gels (Invitrogen, #NP0336BOX and #WG1403BX10), PVDF membrane (Millipore, #IPVH85R), and TPBS with 5% milk powder (Cell Signaling, #9999) were used. The primary antibodies used in this study were: mouse antibody against Calnexin (BD Biosciences, #610524; RRID:AB_397884), against CD63 (H5C6; Novus, #NBP2-42225; RRID:AB_2884028), against CD81 (JS81; BD Biosciences, #555675; RRID:AB_396028), against CD9 (Clone 209306; R&D Systems, #MAB1880; RRID:AB_2075900), against Flotillin-1 (BD Biosciences, #610821; RRID:AB_398140), against GRP78 (BD Biosciences, #610978; RRID:AB_398291), against Rab8 (clone 4; BD Biosciences, #610844; RRID:AB_398164), against TSG101 (Clone 51; BD Biosciences, #612697; RRID:AB_399937), and against TUBB3 (6G7; Developmental Studies Hybridoma Bank, # RRID: AB_528497); rabbit antibody against Caveolin (Abcam, #ab2910; RRID:AB_303405), against COX IV (3E11; Cell Signaling, #4850; RRID:AB_2085424), and against NSE (Abcam, #ab79757; RRID:AB_1603753). The secondary antibodies were goat anti-mouse (Abcam, #ab205719; RRID:AB_2755049) and goat anti-rabbit (Thermo Fisher, #31460; RRID:AB_228341).
